# Attention, in and Out: Scalp-Level and Intracranial EEG Correlates of Interoception and Exteroception

**DOI:** 10.3389/fnins.2017.00411

**Published:** 2017-07-19

**Authors:** Indira García-Cordero, Sol Esteves, Ezequiel P. Mikulan, Eugenia Hesse, Fabricio H. Baglivo, Walter Silva, María del Carmen García, Esteban Vaucheret, Carlos Ciraolo, Hernando S. García, Federico Adolfi, Marcos Pietto, Eduar Herrera, Agustina Legaz, Facundo Manes, Adolfo M. García, Mariano Sigman, Tristán A. Bekinschtein, Agustín Ibáñez, Lucas Sedeño

**Affiliations:** ^1^Laboratory of Experimental Psychology and Neuroscience, Institute of Cognitive and Translational Neuroscience, INECO Foundation, Favaloro University Buenos Aires, Argentina; ^2^National Scientific and Technical Research Council Buenos Aires, Argentina; ^3^Instituto de Ingeniería Biomédica, Facultad de Ingeniería, Universidad de Buenos Aires Buenos Aires, Argentina; ^4^Programa de Cirugía de Epilepsia, Hospital Italiano de Buenos Aires Buenos Aires, Argentina; ^5^Pontificia Universidad Javeriana Bogotá, Colombia; ^6^Centro de Memoria y Cognición Intellectus Bogotá, Colombia; ^7^Unit of Applied Neurobiology, Centro de Educación Médica e Investigaciones Clínicas Norberto Quirno, Consejo Nacional de Investigaciones Científicas y Técnicas Buenos Aires, Argentina; ^8^Departamento de Estudios Psicológicos, Universidad ICESI Cali, Colombia; ^9^Australian Research Council, Centre of Excellence in Cognition and its Disorders, Macquarie University Sydney, NSW, Australia; ^10^Faculty of Education, National University of Cuyo Mendoza, Argentina; ^11^Laboratory of Neuroscience, Universidad Torcuato Di Tella Buenos Aires, Argentina; ^12^Departamento de Fısica, Facultad de Ciencias Exactas y Naturales, Universidad de Buenos Aires and Instituto de Fısica de Buenos Aires, Consejo Nacional de Investigaciones Científicas y Técnicas Buenos Aires, Argentina; ^13^Department of Psychology, University of Cambridge Cambridge, United Kingdom; ^14^Center for Social and Cognitive Neuroscience, School of Psychology, Universidad Adolfo Ibáñez Santiago, Chile; ^15^Universidad Autónoma del Caribe Barranquilla, Colombia

**Keywords:** interoception, exteroception, interoceptive learning, heart evoked potential, intracranial recordings

## Abstract

Interoception, the monitoring of visceral signals, is often presumed to engage attentional mechanisms specifically devoted to inner bodily sensing. In fact, most standardized interoceptive tasks require directing attention to internal signals. However, most studies in the field have failed to compare attentional modulations between internally- and externally-driven processes, thus probing blind to the specificity of the former. Here we address this issue through a multidimensional approach combining behavioral measures, analyses of event-related potentials and functional connectivity via high-density electroencephalography, and intracranial recordings. In Study 1, 50 healthy volunteers performed a heartbeat detection task as we recorded modulations of the heartbeat-evoked potential (HEP) in three conditions: exteroception, basal interoception (also termed interoceptive accuracy), and post-feedback interoception (sometimes called interoceptive learning). In Study 2, to evaluate whether key interoceptive areas (posterior insula, inferior frontal gyrus, amygdala, and somatosensory cortex) were differentially modulated by externally- and internally-driven processes, we analyzed human intracranial recordings with depth electrodes in these regions. This unique technique provides a very fine grained spatio-temporal resolution compared to other techniques, such as EEG or fMRI. We found that both interoceptive conditions in Study 1 yielded greater HEP amplitudes than the exteroceptive one. In addition, connectivity analysis showed that post-feedback interoception, relative to basal interoception, involved enhanced long-distance connections linking frontal and posterior regions. Moreover, results from Study 2 showed a differentiation between oscillations during basal interoception (broadband: 35–110 Hz) and exteroception (1–35 Hz) in the insula, the amygdala, the somatosensory cortex, and the inferior frontal gyrus. In sum, this work provides convergent evidence for the specificity and dynamics of attentional mechanisms involved in interoception.

## Introduction

Interoception can be broadly defined as the sensing of our internal bodily signals (Craig, 2002, 2009; Critchley et al., 2004). This complex process involves multiple dimensions (Garfinkel et al., 2015), such as the conscious perception of visceral information and learning about the underlying mechanisms (Melloni et al., 2013; Couto et al., 2014; Sedeno et al., 2014; Canales-Johnson et al., 2015; Yoris et al., 2015; García-Cordero et al., 2016). The neural basis of interoception spans various regions, crucially including the anterior cingulate cortex, the insular cortex, the inferior frontal gyrus, and the sensorimotor cortex (Craig, 2002, 2009; Critchley et al., 2004; Becker et al., 2015; García-Cordero et al., 2016; Hassanpour et al., 2016; Pollatos et al., 2016; Schulz, 2016; Strigo and Craig, 2016). These areas present multiples connections to the amygdala, the hypothalamus, and the hippocampus, and their interaction yields an integrated mapping of the individual's physiological state (Critchley et al., 2002; Craig, 2009; Becker et al., 2015; Kleint et al., 2015).

Key insights into the neural dynamics of interoception come from the heartbeat evoked potential (HEP), an electrophysiological component associated to afferent cardiac information (Schandry et al., 1986; Montoya et al., 1993; Pollatos et al., 2005, 2016). HEP modulations reflect not only heartbeat perception (Pollatos and Schandry, 2004), but also other processes related to body-brain communication, such as body awareness (Muller et al., 2015), emotional experience (Couto et al., 2015b), motivation (Weitkunat, 1990), attention (Montoya et al., 1993), pain perception (Shao et al., 2011), and stress (Gray et al., 2007). Given these properties, measurements of the HEP may afford critical insights into the cortical monitoring of internal signals, potentially shedding light on two relevant yet underexplored issues: (i) the specificity of internally driven (relative to externally driven) attentional processes; and (ii) its susceptibility to interoceptive training. A systematic assessment of such matters could afford valuable normative or reference parameters for clinical research, given that the HEP alterations during interoceptive tasks have been reported in patients with various psychiatric and neurological conditions (Terhaar et al., 2012; Muller et al., 2015; Schulz et al., 2015; García-Cordero et al., 2016). To foster progress in this direction, here we report the first joint assessment of the two abovementioned issues through a combination of behavioral measures, high-density electroencephalography (hd-EEG), and intracranial recordings.

So far, only a few studies have assessed differences in HEP modulation triggered by internal and external stimuli, yielding mixed results. Some of them reported an increased HEP modulation when attention was focused on heartbeats (Schandry et al., 1986; Montoya et al., 1993; Schandry and Montoya, 1996), suggesting that this component may index distinctively interoceptive processes. Conversely, other experiments showed no differential modulations between conditions (Terhaar et al., 2012). In fact, knowing whether exteroceptive and interoceptive processing rely on different or shared mechanisms could contribute to our understanding of body-brain interactions in healthy subjects and in neuropsychiatric disorders. However, the vast majority of interoceptive research in neurotypicals (Pollatos and Schandry, 2004; Pollatos et al., 2005; Canales-Johnson et al., 2015) (except for Montoya et al., 1993) and patients with neuropsychiatric diseases (Terhaar et al., 2012; Muller et al., 2015; Schulz et al., 2015; García-Cordero et al., 2016), has overlooked contrasts between interoceptive and exteroceptive conditions. Thus, the ensuing results do not establish whether the observed effects reflect modulations of general attentional mechanisms or the specific dynamics of internally-driven processes. Here, we aim to directly address this issue by combining methods with high temporal and spatial resolution.

Second, evidence on interoceptive learning is also scant (Schandry and Weitkunat, 1990; Melloni et al., 2013; Sedeno et al., 2014; Canales-Johnson et al., 2015; Yoris et al., 2015; García-Cordero et al., 2016). Studies on motor-based heartbeat tracking show that exposure to feedback or training consistently improves interoceptive performance (Melloni et al., 2013; Couto et al., 2014; Yoris et al., 2015; García-Cordero et al., 2016), with about half the participants showing no learning (Canales-Johnson et al., 2015). Importantly, the only work reporting null behavioral effects of training (Schandry and Weitkunat, 1990) measured interoceptive accuracy through a radically different paradigm—a heartbeat discrimination task, in which participants had to identify whether auditory tones were in synchrony with their own heartbeats. Interoceptive learning has also been associated with distinct HEP modulations, namely, increased cortical activity to cardiac signals (Schandry and Weitkunat, 1990) and enhanced negativity in the post-feedback condition (Canales-Johnson et al., 2015). Moreover, imaging results suggest that interoceptive learning seems to rely on fronto-temporal network connectivity (García-Cordero et al., 2016), involving key substrates of general learning, memory and multimodal association processes. Compatibly, metacognitive awareness of learning seems mediated by the synchronization of long-range fronto-temporal signals (Canales-Johnson et al., 2015).

Despite their contributions, the studies reviewed above feature important limitations. First, those assessing differences between internal and external attention included relatively small sample sizes (fewer than 30 subjects) and employed low-density EEG recordings (Schandry et al., 1986; Montoya et al., 1993; Schandry and Montoya, 1996)—the only exception being (Terhaar et al., 2012), who nevertheless failed to consider specific scalp regions. Moreover, none of them combined scalp and intracranial recording to tap anatomo-functional differences between external and internal attention. Second, previous works on interoceptive learning evaluated electrophysiological modulations considering only between-group comparisons (good learners vs. bad learners, or trained vs. untrained subjects) (Schandry and Weitkunat, 1990; Canales-Johnson et al., 2015). The HEP differences reported therein were thus associated with individual performance, which prevents assessing whether learning involves basic modulation changes irrespective of behavioral learning scores. Therefore, it remains unclear whether interoceptive learning is related to changes in the HEP when considering a single sample of healthy subjects. In addition, no previous study has compared HEP modulations in post-feedback interoception vs exteroception.

To address these issues, we evaluated a large sample of healthy subjects with a validated heartbeat detection (HBD) task. We assessed three conditions: exteroception, basal interoception, and post-feedback interoception, while measuring HEP modulations. Then, to better understand the ongoing functional dynamics of interoceptive hubs and its specificity in interoceptive processes, we evaluated exteroception and basal interoception in two epileptic patients featuring depth electrodes in key interoceptive areas. Importantly, this multimethodological approach enabled us to explore differential brain connectivity patterns and modulations with great spatiotemporal precision.

In light of previous findings, we hypothesized that (a) electrophysiological dynamics (HEP and intracranial recordings) would distinguish interoceptive from exteroceptive processes, and (b) HEP modulations would be greater for both interoceptive conditions (before and after feedback) than exteroceptive processes. In addition, we explored whether interoception involved distinctive connectivity properties among critical hubs.

## Materials and methods

### Participants

#### Healthy subjects

Study 1 comprised 50 healthy volunteers (32 female) between 19 and 67 years old (*M* = 36.5, *SD* = 12.63) with an average of 17.02 years of education (*SD* = 2.64). All of them underwent a standard clinical examination including extensive neurological, neuropsychiatric, and neuropsychological assessments. Results from the INECO Frontal Screening battery (Torralva et al., 2009) revealed normal executive function scores (*M* = 27.15, *SD* = 1.74) over a maximum of 30 points. None of the subjects had a history of neurologic or psychiatric disorders, substance abuse or heart disease. All participants read and signed a written consent that stipulates the details of the study and allows for its publication. This study was carried out in accordance with the recommendations of the Hospital Italiano and INECO committee with written informed consent from all subjects. All subjects gave written informed consent in accordance with the Declaration of Helsinki. The protocol was approved by the Ethics Committee for Research Protocols of the Italian Hospital University and the Institutional Committee of Ethics of the Institute of Cognitive Neurology.

#### Intracranial patients

In Study 2, we profited from access to two male patients with depth electrodes implanted in key interoceptive regions. Intracranial recordings are a unique method to assess human neurocognition with high spatial and temporal resolution (Jacobs and Kahana, 2010; Dastjerdi et al., 2013; Parvizi et al., 2013; Musch et al., 2014; Foster et al., 2015; Noy et al., 2015), as shown in previous studies assessing emotions (Ponz et al., 2014; Hesse et al., 2016), cognition (Jensen et al., 2002; Meltzer et al., 2008; Noy et al., 2015), and, more particularly, interoception (Canales-Johnson et al., 2015). In our case, the two patients suffered from pharmacologically intractable epilepsy and were undergoing presurgical evaluation. Patient 1 (P1) is a 33-year-old right-handed undergraduate who had been suffering from epileptic crises since age four. He had depth electrodes in right frontal, parietal and temporal regions, including the posterior insula (*n* = 3), the amygdala (*n* = 3), and the somatosensory cortex (*n* = 2). Patient 2 (P2) is a 35-year-old right-handed man suffering from epileptic crises since the age of 14. He had depth electrodes in the left inferior frontal gyrus, pars opercularis (*n* = 3). Participants read and signed a written consent that stipulates the details of the study and allows for its publication. This study was carried out in accordance with the recommendations of the Hospital Italiano and INECO committee with written informed consent from all subjects. All subjects gave written informed consent in accordance with the Declaration of Helsinki. The protocol was approved by the Ethics Committee for Research Protocols of the Italian Hospital University and the Institutional Committee of Ethics of the Institute of Cognitive Neurology.

### HBD task

We used a validated HBD task (Melloni et al., 2013; Couto et al., 2014, 2015a; Sedeno et al., 2014; Canales-Johnson et al., 2015; Yoris et al., 2015, 2017; García-Cordero et al., 2016) in which participants are required to tap a computer keyboard along with their heartbeats or external stimuli. First, in the exteroceptive condition, participants were instructed to follow an audio recording of a simulated heartbeat for 2 min. This was done twice. The first time, beats were presented at a constant frequency (60 bpm); the second time, their frequency was inconsistent and variable. Next, in the basal interoception condition, subjects were asked to follow their own heartbeats in the absence of any sensory feedback for 2 min. This condition provides an objective measure of each participant's interoceptive accuracy at baseline (Garfinkel et al., 2015). This was also performed twice. Then, subjects listened to their own heartbeats through a stethoscope and tracked them by tapping on the keyboard for a block of 2 min. This was aimed to give participants feedback on their real heartbeats to improve performance in the following condition (hence, this part of the task is not subject to analysis). As previous works have shown, only one block of feedback is necessary to induce interoceptive improvements (Melloni et al., 2013; Sedeno et al., 2014; Canales-Johnson et al., 2015; Yoris et al., 2015; García-Cordero et al., 2016). Finally, in post-feedback interoception, participants were asked to follow their heartbeats again in the absence of any sensory feedback, which offered a measure of learning from previous feedback. The accuracy index was calculated for every condition on a scale ranging from 0 to 1, with higher scores indicating better performance (Melloni et al., 2013; Sedeno et al., 2014; Couto et al., 2015a; Yoris et al., 2015; García-Cordero et al., 2016)—see Supplementary Material 1.2.1. Given that the HBD task requires at least 40–60 trials for reliable estimations (Kleckner et al., 2015), we set a very robust regime, considering four times this standard value of trials, except for the feedback condition (exteroception: *M* = 287.04; basal interoception: *M* = 269.9; feedback: *M* = 130.54; post-feedback interoception: *M* = 260.24). During the HBD task, we recorded hd-EEG and electrocardiographic (ECG) signals, as detailed below.

### Data recording

#### Study 1

Hd-EEG and ECG signals were sampled at 1,024 Hz, with a Biosemi Active Two 128-channel system and two Ag/Ag-Cl adhesive electrodes placed in lead-II positions. Data were recorded while the subjects performed the HBD task.

#### Study 2

Recordings were obtained from two epileptic patients, as done in previous research (Jacobs and Kahana, 2010; Ibanez et al., 2013; Canales-Johnson et al., 2015; Foster et al., 2015; Hesse et al., 2016), while they completed the first two parts of the HBD task (exteroception and basal interoception). To meet the strict time constraints of intracranial protocols and avoid discomfort and fatigue in the participants, in this study we used an abridged version of the HBD task. Data were derived from semi-rigid, multi-lead electrodes and sampled at 1,024 Hz. The electrodes had a diameter of 0.8 mm and consisted of 5, 10, or 15 2-mm-wide contact leads placed 1.5 mm apart from each other (DIXI Medical Instruments). The video-SEEG monitoring system (Micromed) recorded depth-EEG electrode sites simultaneously. The two patients had a total of 128 contact sites in frontal, parietal, and temporal regions. All task-relevant regions were distant from epileptogenic foci, and no recording site presented epileptogenic activity or within dysplasia regions. Moreover, we established stringent inclusion criteria for the remaining channels (Manning et al., 2009; Dastjerdi et al., 2013; Foster et al., 2015; Hesse et al., 2016), and subjected MRI scans to careful inspection to rule out structural abnormalities (see details in Data Processing section of Study 2).

For our analysis we targeted electrodes located in key interoceptive regions as established through MRI, stimulation protocols, source analysis, and previous intracranial recordings. These included the posterior insula (Craig et al., 2000; Kong, 2006; Craig, 2009; Hassanpour et al., 2016; Kuehn et al., 2016; Schulz, 2016), the inferior frontal gyrus (Pollatos et al., 2007; Zaki et al., 2012; Kuehn et al., 2016), the somatosensory cortices (Craig, 2002; Critchley et al., 2004; Pollatos et al., 2005, 2016; Khalsa et al., 2009), and the amygdala (Critchley et al., 2002; Becker et al., 2015; Kleint et al., 2015). Although the latter area is not so widely recognized for its role in interoception, it has structural connections with the insula (Mesulam and Mufson, 1982) and it cooperates with the anterior cingulate cortex and the prefrontal cortices in the construal of interoceptive states (Craig, 2002; Critchley, 2005; Garfinkel and Critchley, 2016) (see **Figure 4A** and Supplementary Table 1 for details about electrode localization).

Following previous works of our group (Chennu et al., 2013; Ibanez et al., 2013; Canales-Johnson et al., 2015), the electrodes' spatial locations were obtained with post implantation MRI and CT scans from both patients. Both volumetric images were affine registered and normalized using SPM8 Matlab toolbox (Friston, 2007). The MNI coordinates and Brodmann areas of each contact site were obtained using MRIcron (Rorden and Brett, 2000). To define the patients' results in a common space and enhance their visualization, we used the normalized position of the electrodes' contact sites to a MNI coordinate space.

### Data processing

#### Study 1

##### Heartbeat evoked potential

Data were referenced offline to mastoids and then resampled to 256 Hz and band-pass filtered (low: 0.5; high: 30 Hz) to remove undesired frequency components. To analyze HEP modulations, we used R-wave-ECG detection values to segment each of the 128 channels of the EEG data. R-wave-ECG detection was achieved through the PeakFinder function implemented in Matlab, which quickly finds local peaks or valleys (local extrema) in a noisy vector, using the alternating nature of the derivatives along with a user-defined magnitude threshold to determine whether each peak is significantly larger (or smaller) than the data around it (Kruczyk et al., 2013). As the cardiac field artifact (CFA) (Kern et al., 2013) and the cortical signal of interest (HEP) are time-locked to the same event and thus spatio-temporally overlapped, we removed CFA contamination using visual inspection and Independent Component Analysis (ICA) (Kim and Kim, 2012), considering thresholds of ±300 μV. To this end, we explored in every subject those components that showed a higher voltage coinciding with the heart signal R-wave as well as a greater posterior positivity and greater anterior negativity on the component topography, as shown in Terhaar et al. (2012). Finally, we visually compared the HEP before and after removing the CFA so as to assess the effect of the artifact removal on the cardiac potential. In addition, eye-movement contamination was removed using visual inspection and ICA. These approaches have been used in previous studies to obtain reliable HEPs (Dirlich et al., 1997; Pollatos and Schandry, 2004; Terhaar et al., 2012; García-Cordero et al., 2016). Then, the data were segmented from 200 ms prior to the R-wave-ECG onset to 500 ms after its onset. Noisy epochs were rejected from the analysis using a visual procedure.

##### Functional connectivity analysis

We explored brain dynamics in basal and post-feedback interoception using Weighted Symbolic Mutual Information (wSMI), a novel measure of integration and global broadcasting of information across distant cortical regions (King et al., 2013)—for details, see Supplementary Material 1.2.2. The wSMI measure assesses the extent to which two signals present non-random joint fluctuations (sharing information), characterized by (a) fast and robust estimation of the signals' entropies, (b) detection of nonlinear coupling, and (c) absence of spurious correlations between signals arising from common sources (King et al., 2013). This method proves highly sensitive to assess functional connectivity based on intracranial recordings (Hesse et al., 2016), and it allows diminishing the noise caused by common sources and volume conduction, since it does not consider when the information sharing is similar between two signals (King et al., 2013). First, we applied the current source density method (King et al., 2013), which involves subtracting, from each channel, the activity of neighboring sensors; this technique thus diminishes volume conduction and increases the spatial focalization of EEG information. Then, EEG signals were first transformed into a series of discrete symbols defined by the ordering of k time samples segregated by a temporal separation τ. Analysis was restricted to a fixed symbol size (*k* = 3) and a single value of τ (τ = 16 ms, approximately 10–20 Hz). To calculate wSMI for each pair of transformed EEG signals, we estimated the joint probability of each pair of symbols. To reduce spurious correlations between signals, the joint probability matrix was multiplied by binary weights. The weights were set to zero for pairs of identical symbols, which could be elicited by a unique common source, and for opposed symbols, which could reflect the two sides of a single electric dipole. In sum, the wSMI measure is an index calculated from the functional coupling (i.e., information sharing) between two signals, which generates an association matrix for each pair of electrodes per subject (King et al., 2013). Based on these matrices, we performed two connectivity analyses.

First, we established a seed based on a ROI composed of electrodes from the HEP maxima of the previous right-ROI and adjacent Biosemi electrodes (62–69 and 73–77), which also aligned with previous evidence of greater HEP amplitudes in right frontal electrodes (Schandry and Montoya, 1996; Pollatos and Schandry, 2004) and right-sided modulations during body signal processing (Naver et al., 1996; Leopold and Schandry, 2001; Meyer et al., 2004; Couto et al., 2015a; **Figure 3A**, ROI marked in gray). Then, we compared the wSMI matrix between the electrodes inside the ROI and the rest of the scalp. This allows identifying global brain connectivity differences between basal and post-feedback conditions.

Second, to better quantify the possible differences in short-, medium-, and long-range connections between conditions from the first analysis, we assessed wSMI as a function of the Euclidian distance. This analysis consisted in the calculation of wSMI for each pair of electrodes between the right-frontal ROI (mentioned above) and the rest of the scalp, ordered by distance. This method allows obtaining the shortest and direct distance between two points (or electrodes) over the scalp surface, and its robustness has been verified in several studies (e.g., King et al., 2013; Hesse et al., 2016). The distance separating EEG channels was calculated along a straight line, using the information provided by the channel location, as done in King et al. (2013) and Hesse et al. (2016):

$$\begin{array}{l} d_{i,j}=\;\sqrt{{\left(x_{i}-x_{j}\right)}^{2}+{\left(y_{i}-y_{j}\right)}^{2}+{\left(z_{i}-z_{j}\right)}^{2}} \end{array}$$

where *d*_*i,j*_ is the distance between the electrode *i* and the *j*, determined by its characteristic Cartesian coordinates *x, y*, and *z*.

#### Study 2

##### Preprocessing of intracranial recordings and time frequency decomposition

Data were bandpass-filtered from 1 to 200 Hz with a zero phase shift finite impulse filter. Then, to eliminate main artifacts, they were notch filtered at 50 Hz and at their harmonic frequencies (100 Hz, 150 Hz) (Chen et al., 2013). In each patient, we discarded all the contact sites in the interoceptively relevant brain regions which presented artifacts and pathological waveforms. This was achieved by visual inspection of the recordings and by application of the following criteria: (1) signal values could not exceed five times the signal mean, and/or (2) consecutive signal samples could not exceed five standard deviations (*SD*) from the gradient's mean (Chen et al., 2013). The remaining sites were referenced to the mean value (the average of the sites per subject were averaged and subtracted from each recording) (Rangarajan et al., 2014). Finally, the data were segmented from 200 ms prior the R-wave-ECG onset to 500 ms after its onset. The epochs were baseline-corrected (baseline: −200 ms to −50 ms) (Szczepanski et al., 2014).

Time-frequency analysis was performed for basal interoception and exteroception by means of a windowed Fourier transform (window length: 250 ms, step 8 ms, window overlap 97%) (Gross, 2014; Musch et al., 2014). Time-frequency charts were normalized to baseline before the stimulus onset. The normalization involved subtracting the baseline average and dividing by the baseline SD on a frequency-by-frequency basis, using a window from −200 to 500 relative to the onset of stimuli (Szczepanski et al., 2014).

In light of the relationship between low frequencies (1–35 Hz) and attentional and exteroceptive sensory activity (Narici et al., 1990; Schurmann and Basar, 1994, 2001; Palva and Palva, 2007; Sadaghiani et al., 2010; Cooper et al., 2016; Pandey et al., 2016), and considering that higher oscillations (35–110 Hz) are strengthened by internal tasks, consciousness, and awareness (Meador et al., 2002; Dressler et al., 2004; Melloni et al., 2007; Wyart and Tallon-Baudry, 2009; Canales-Johnson et al., 2015), we explored two frequency ranges: 1–35 Hz and Broadband (BB) from 35 to 110 Hz. The latter is commonly used in intracranial studies (Hesse et al., 2016). We expected interoceptive and exteroceptive processes to modulate signals at higher frequencies (BB) and at lower bands (1–35 Hz), respectively.

### Statistical analysis

#### Study 1

##### HBD task

Following previous reports (Melloni et al., 2013; Yoris et al., 2015) we used repeated-measures ANOVA with a within-subject factor (the three conditions: exteroception, basal interoception, and post-feedback interoception). Tukey's post hoc analysis was performed to explore the differences among conditions (the alpha level was set at <0.05).

##### HEP

ERP data were compared among conditions using Monte Carlo permutation tests (Manly, 2007) combined with bootstrapping. This simple method, used in previous HEP analyses (Couto et al., 2014, 2015b; Canales-Johnson et al., 2015; García-Cordero et al., 2016; Yoris et al., 2017), offers a straightforward solution for multiple comparison problems and does not depend on Gaussian assumptions about the probability distribution of the data. The data from each comparison between conditions was separately subjected to a random partition and a *t*-value was then calculated. This process was repeated 5000 times to construct the *t*-value distribution under the null hypothesis. Then, the null hypothesis was rejected if an obtained *t*-value was greater than the most extreme 5% of the distribution (e.g., *p* < 0.05, two tailed *t*-test). An additional advantage of this method is that ERP analysis is not based on a time window selected a priori. Instead, this data-driven analysis allowed us to evaluate each point of the signal from 100 to 500 ms, covering the typically HEP latency (Montoya et al., 1993; Pollatos and Schandry, 2004; Canales-Johnson et al., 2015).

For the analysis, we selected a right frontal ROI (Biosemi electrodes 67, 68, 69; **Figure 2**) based on previous reports showing greater HEP amplitudes in right frontal electrodes while subjects paid attention to heartbeats (Schandry et al., 1993; Pollatos and Schandry, 2004; Pollatos et al., 2005; Yoris et al., 2017). Indeed, frontal regions, such as the orbitofrontal cortex, the inferior frontal gyrus, the insula, and the anterior cingulate cortex, have been widely associated with interoception (Craig, 2002, 2009; Critchley et al., 2004; Becker et al., 2015; Hassanpour et al., 2016; Kuehn et al., 2016; Pollatos et al., 2016; Schulz, 2016; Strigo and Craig, 2016). Moreover, some studies suggest that body signal processing is considerably right-lateralized (Naver et al., 1996; Leopold and Schandry, 2001; Meyer et al., 2004; Couto et al., 2015a). However, given that other reports have also shown HEP modulation in central and left central regions (Schandry and Montoya, 1996; Pollatos et al., 2005, 2016; Gray et al., 2007; Shao et al., 2011; Canales-Johnson et al., 2015), we also contemplated a fronto-central (Biosemi electrodes: 81, 82, 83) and a left-frontal (Biosemi electrodes: 99, 100, 101) ROI–**Figure 2**.

##### Statistical analysis of functional connectivity data

In order to analyze connectivity differences between basal and post-feedback interoception, we performed two-tailed *t*-tests between the wSMI matrices of these two conditions. The analysis was performed by comparing both conditions (basal vs. post-feedback interoception), and the *p*-values were set at <0.05 (for exploratory display) and at <0.01 to highlight the strongest connections of the scalp. Also, to investigate the relationship between wSMI and the Euclidian distance among channels in basal and post-feedback interoception, we performed Monte Carlo permutation tests (Manly, 2007) combined with bootstrapping following the procedure explained in the Statistical Analysis section of Study 1 (HEP) (*p* < 0.001, as in King et al., 2013).

#### Study 2

##### Time frequency statistical analysis of intracranial recordings

Statistical analysis of basal interoception and exteroception was performed by means of Monte Carlo permutation tests combined with bootstrapping following the procedure explained in the Statistical Analysis section of Study 1 (HEP) (*p* < 0.05, as in previous reports with intracranial recordings Hesse et al., 2016). Each point of the signal from 100 ms onwards was evaluated with this permutation analysis.

## Results

### Study 1

#### HBD task

**Figure 1:**
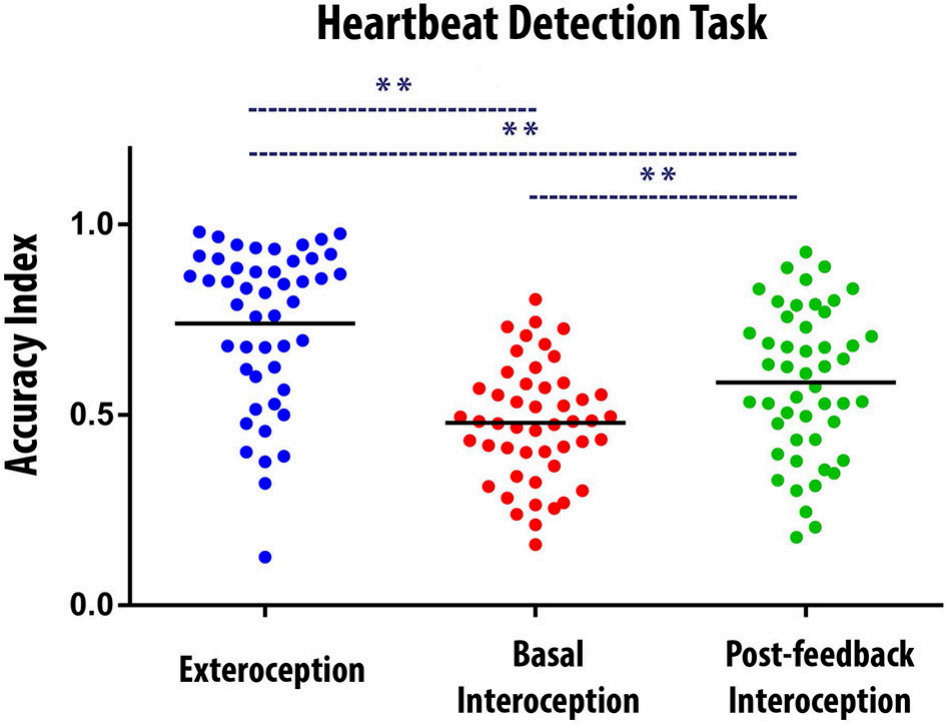
Behavioral results of the Heartbeat Detection (HBD) task. Dots show the mean performance of each subject for each condition (i.e., the average of the two blocks of each condition). The Accuracy Index can vary between 0 and 1, with higher scores indicating better interoceptive accuracy. ^**^ Indicates significant differences between the conditions, set at *p* < 0.05. The black bars indicate the mean of the data distribution.

Overall, participants' performance was better (a) during exteroception than during both interoceptive conditions and (b) in post-feedback interoception, than in basal interoception. This finding was confirmed by an ANOVA with the three conditions as independent factors [*F*_(2, 94)_ = 31.95; *p* < 0.001; exteroceptive condition: *M* = 0.73, *SD* = 0.20; basal interoception: *M* = 0.47, *SD* = 0.15; post-feedback interoception: *M* = 0.58, *SD* = 0.19]. A Tukey post hoc analysis (*MS* = 0.025; *df* = 94) indicated that performance was better when subjects were following external stimuli than when they followed their own heartbeats (exteroception vs. basal interoception: *p* < 0.001, and exteroception vs. post-feedback interoception: *p* < 0.001). In addition, participants performed better in the post-feedback interoception than in the basal interoception condition (*p* < 0.01). This result shows that participants learned during the feedback condition and that this learning was sufficiently effective to increase HBD accuracy after this condition (Figure 1).

### ERP results: the HEP among conditions

**Figure 2:**
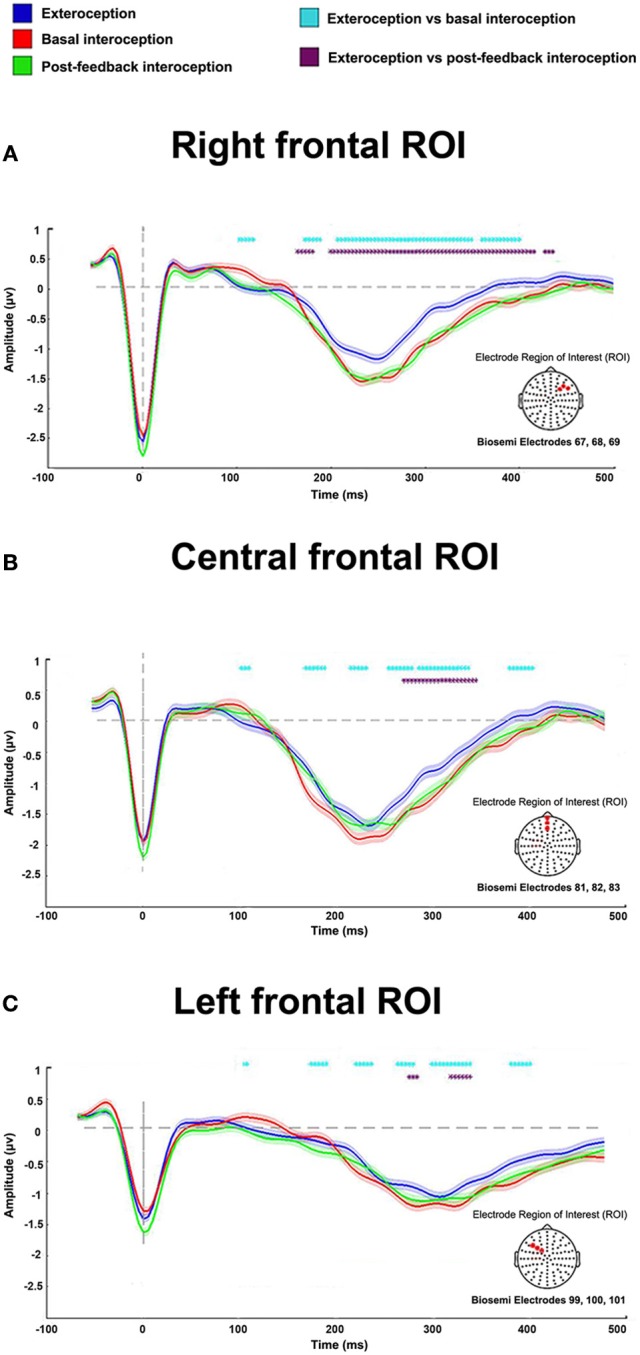
HEP differences among conditions for the right, central, and left ROIs. Results show an enhanced modulation in all ROIs of the interoceptive conditions (basal and post-feedback) compared to the exteroceptive one. Dotted marks identify significant values between conditions (*p* < 0.05). Differences not reaching five consecutive points were eliminated to avoid noisy results (i.e., differences that were only for one time point but not sustained in time, as done in previous reports, Couto et al., 2015b; García-Cordero et al., 2016); shadows indicate standard error of the mean (SEM).

In the right frontal ROI, HEP modulations were significantly more negative for the interoceptive conditions (basal and post-feedback) compared to the exteroceptive one within the expected time-window (200 to 500 ms). Bootstrapped permutations showed that, relative to the exteroceptive condition, basal interoception presented an enhanced modulation from 171 to 187 ms, 207 to 347 ms, and 359 to 398 ms (*p* < 0.05), and post-feedback interoception, at 164 to 179 ms and 199 to 414 ms (*p* < 0.05) (Figure 2A). Similar modulations were found in the central frontal and left frontal ROIs, which further strengthens our findings. For the central frontal ROI, relative to exteroception, basal interoception presented greater HEP modulations from 168 to 187 ms, 214 to 230, 253 to 277 ms, 285 to 335 ms, and to 378 to 402 ms (*p* < 0.05); and post-feedback interoception, from 269 to 343 ms (*p* < 0.05) (Figure 2B). Finally, for the left ROI, HEP modulation for basal interoception was enhanced from 218 to 237 ms, 261 to 277 ms, 296 to 335 ms, and 378 to 398 ms (*p* < 0.05); and post-feedback interoception, from 273 to 281 ms and 312 to 335 ms —all relative to exteroception (Figure 2C).

#### Functional connectivity results

**Figure 3:**
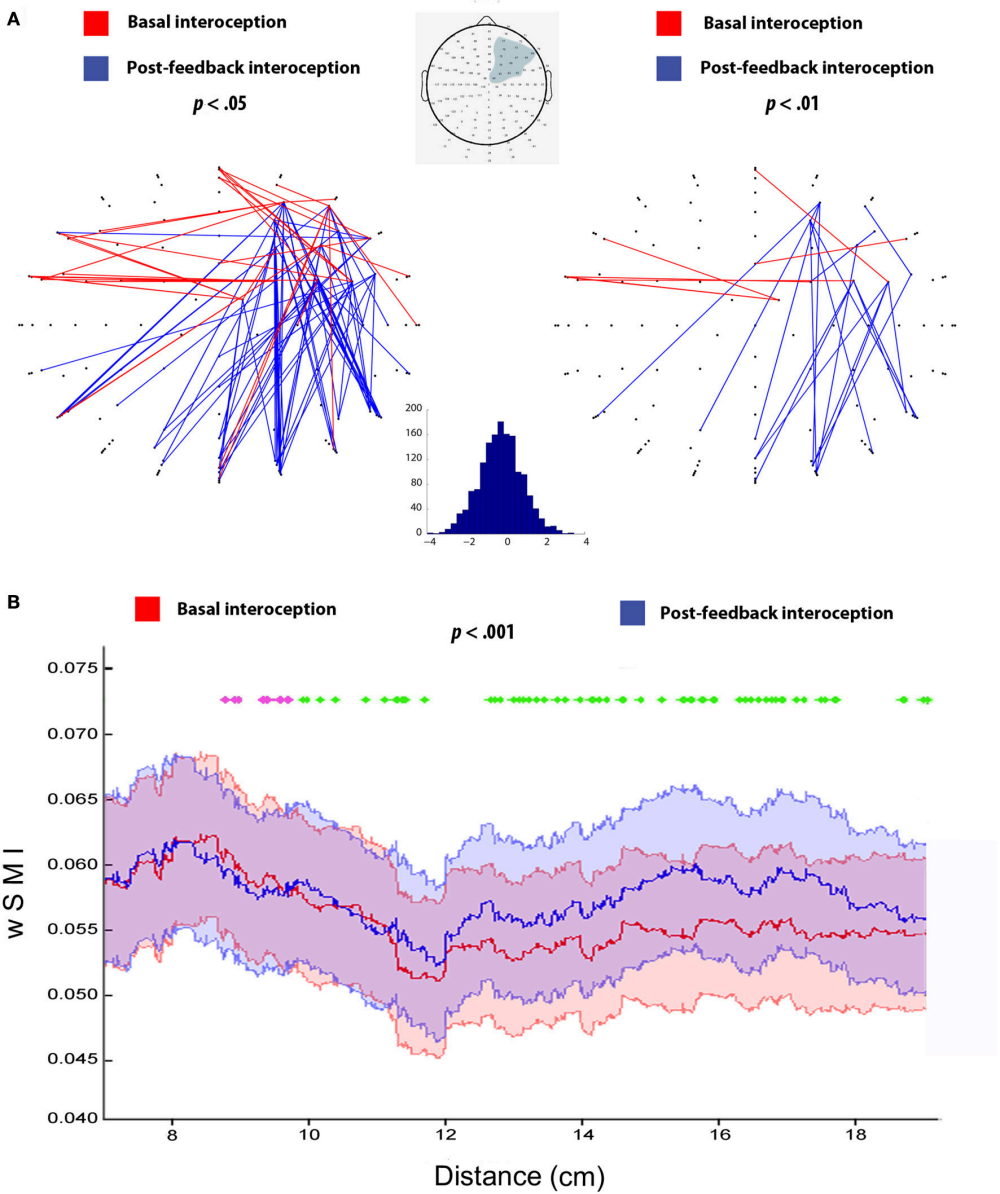
**(A)** Connectivity analysis for basal and post-feedback interoception at *p* < 0.05 and *p* < 0.01, following previous studies (Canales-Johnson et al., 2015). Red lines indicate connections that are significantly higher for basal interoception and blue lines represent enhanced connections for post-feedback interoception, both at the 10–20 Hz frequency band (τ = 16 ms). The gray shadow in the scalp diagram delimits the selected ROI used for the analysis. The histogram indicates *t*-values distribution from the comparison between basal (>0) and post-feedback interoception (<0). **(B)** wSMI as a function of inter-channel distance for basal and post-feedback condition (*p* < 0.001, as in King et al., 2013). Pink dotted lines show differences in favor of basal interoception for short distance connectivity, while green dotted lines indicate significant differences in favor of post-feedback interoception for long range connectivity; (*p* < 0.001). The X axis shows Euclidian distance in cm based on Channel Location coordinates (King et al., 2013). The color shadows plotted indicates the SEM of each condition.

Figure 3A shows connectivity differences between conditions, with two different statistical thresholds (two-tailed *t*-tests, *p*-values < 0.05 and < 0.01). This analysis revealed enhanced local connectivity for basal interoception, particularly distributed across right-sided and bilateral fronto-central regions of the scalp. Conversely, post-feedback interoception featured more distributed connectivity, comprising electrodes from frontal and posterior areas.

This differential pattern of more frontal vs. more posterior connectivity was further supported by the analysis of the wSMI as a function of distance, which objectively measures differences in short-, medium-, and long- distances. Specifically, we observed that basal interoception presented an increased connectivity values in short-distance connections compared to post-feedback interoception (from approximately 8–10 cm, *p* < 0.001). On the other hand, we found that connectivity values were higher in the long-range connections for post-feedback interoception compared to basal interoception (from approximately 10 to 18 cm, *p* < 0.001; Figure 3B).

### Study 2

#### Intracranial results of interoceptive-exteroceptive processing

**Figure 4:**
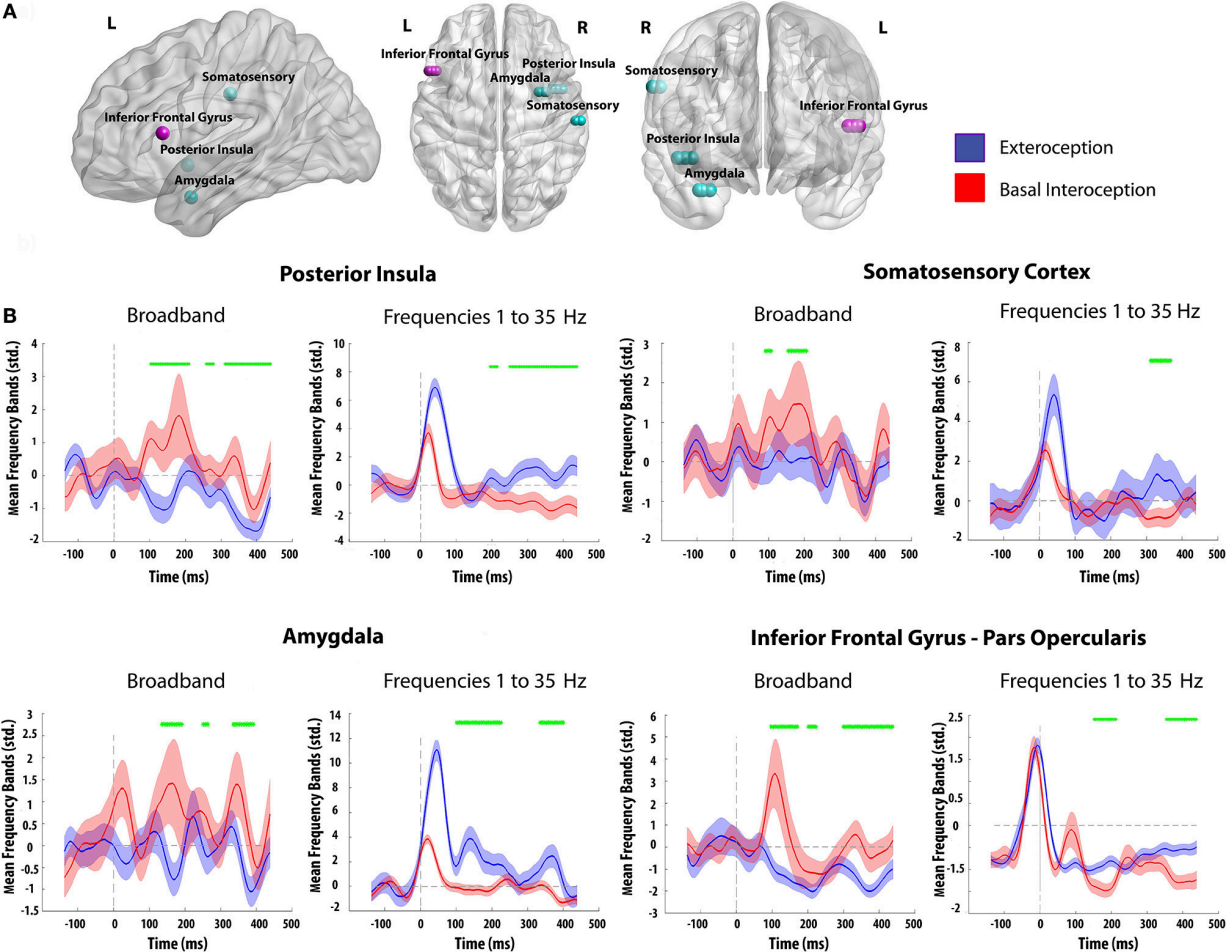
**(A)** Electrode contact sites of both subjects. Each color represents a different patient (Light Blue: Patient 1; Pink: Patient 2). **(B)** Time frequency analysis for basal interoception and the exteroceptive condition using different frequency ranges. Green marks identify significant values between conditions (*p* < 0.05, as in Hesse et al., 2016). Differences not reaching five consecutive points were eliminated; shadows indicate SD.

Time-frequency analysis showed differences in the frequency bands between interoceptive and exteroceptive conditions. When measuring the BB frequencies (35–110 Hz), significant differences (*p* < 0.05) were found in favor of basal interoception on the four selected regions (posterior insula: from 100 to 209 ms, 258 to 278 ms, and 313 to 437 ms; amygdala: from 100 to 192 ms, 249 to 264 ms, and 333 to 391 ms; somatosensory cortex: from 100 to 108 ms, and 153 to 206 ms; inferior frontal gyrus: from 100 to 171 ms, 200 to 223 ms, and 298 to 437 ms). In contrast, an inverse pattern of modulation was found at lower frequencies (1–35 Hz), favoring the exteroceptive condition (posterior insula: from 195 to 214 ms, and 249 to 437 ms; amygdala: from 100 to 226 ms, and 333 to 400 ms; somatosensory cortex: from 310 to 368 ms; inferior frontal gyrus: from 151 to 212, and 353 to 437 ms) (Figure 4B).

## Discussion

Through a combination of behavioral and electrophysiological measures, this study aimed to provide new insights into (i) the functional distinctiveness of interoceptive relative to exteroceptive processes, and (ii) the susceptibility of interoceptive mechanisms to body-signal feedback. First, we found that differential attentional mechanisms for internal and external signals were indexed by HEP modulations. Relative to exteroception, interoception (both before and after feedback), yielded greater HEP modulations and differential oscillatory dynamics within key brain regions. Also, behavioral differences between basal and post-feedback interoception were mirrored only by their connectivity patterns: whereas basal interoception was characterized by local connectivity involving short-range connections, post-feedback interoception presented long-range connections and a widespread connectivity pattern, involving frontal and posterior regions. These findings contribute to our understanding of the complexities of interoception, and afford a set of parameters against which to interpret potential interoceptive abnormalities in pathological conditions.

### Attentional mechanisms specific to interoception

HEP modulations were significantly more negative for interoceptive conditions (basal and post-feedback) than for the exteroceptive one in the three frontal ROIs. In line with this result, several studies have reported HEP modulations, not only in right electrodes (Pollatos and Schandry, 2004), but also extended to left (Schandry and Weitkunat, 1990; Gray et al., 2007) and central (Schandry and Weitkunat, 1990; Pollatos and Schandry, 2004; Schulz et al., 2015) topographies. Thus, it would seem that interoception and exteroception imply different neural mechanisms indexing attentional processes at very fast ongoing cycles.

Previous studies show that attention toward internal stimuli, relative to external ones, yields different fronto-central HEP modulations (Schandry et al., 1986; Montoya et al., 1993; Schandry and Montoya, 1996). Moreover, this modulation seems greater in poor interoceptive perceivers, arguably reflecting increased attentional effort to perceive internal signals (Montoya et al., 1993). Taken together, the evidence suggests that, HEP measurements based on the present protocol may be sensitive to attentional allocation toward internal sensations regardless of detection accuracy.

This functional distinction between interoceptive and exteroceptive mechanisms was corroborated and anatomically refined by the results from our second study. Activity in critical hubs of interoception (insula, amygdala, somatosensory cortex, and inferior frontal gyrus) was maximal in higher frequencies for interoceptive activity and in lower frequencies for exteroceptive processes. Whereas none of the available interoceptive studies with intracranial recordings have considered differences between interoceptive and exteroceptive conditions or analyzed ongoing time-frequency patterns (Kern et al., 2013; Canales-Johnson et al., 2015; Babo-Rebelo et al., 2016), recent works have related oscillatory activity in higher range bands related to awareness (Dressler et al., 2004) and second-order functions such as directed attention, memory, and learning (Kaiser and Lutzenberger, 2003; Herrmann et al., 2004; Jensen et al., 2007). Moreover, accruing evidence attests to the role of lower bands in exteroceptive and unimodal processes (Narici et al., 1990; Roth and Sack, 1990; Schurmann and Basar, 1994, 2001; Palva and Palva, 2007; Sadaghiani et al., 2010). Accordingly, exteroception may be proposed to involve less demanding perceptual operations, with subjects relying on well-developed sensory skills to follow external cues. In contrast, interoception would prove more cognitively demanding, as attention and sensation must be directed inwards and thus rely on less trained mechanisms, resulting in higher uncertainty. This interpretation accounts for the oscillatory-band differences observed in Study 2, highlighting the functional distinctiveness of interoceptive vs. exteroceptive attentional mechanisms. Moreover, by offering specific neuroanatomical foundations for these differential dynamics, our results refine extant models of interoceptive brain networks (Kleint et al., 2015; García-Cordero et al., 2016).

Regarding the electrophysiological differences between interoceptive and exteroceptive processes, our study presents complementary methodological advances in comparison to previous reports (Schandry et al., 1986; Montoya et al., 1993; Schandry and Montoya, 1996; Terhaar et al., 2012), such as the inclusion of a large sample size (50 participants), the combined analysis of hd-EEG and iEEG recordings, and the use of a statistical approach that avoids the potential bias of selecting a-priori time windows (point-by-point permutation tests). Based on these methodological foundations, our scalp-level and intracranial electrophysiological recordings converged in showing specific cortical mechanism for attention to internal-driven stimuli compared to external ones.

### Neural bases of interoceptive learning

Behaviorally, participants performed better in post-feedback interoception relative to basal interoception. This result replicates previous findings of our group (Melloni et al., 2013; Couto et al., 2014; Sedeno et al., 2014; Canales-Johnson et al., 2015; Yoris et al., 2015; García-Cordero et al., 2016), showing that the monitoring of visceral signals can be enhanced through deliberate training.

Somewhat surprisingly, the above pattern was not accompanied by differential HEP modulations. In a previous study, Schandry and Weitkunat (1990) reported increased cortical activity in a post-training condition, which they associated with enhanced sensitivity to cardiac signals. Greater HEP modulations, accompanying enhanced post-feedback performance, were also reported by Canales-Johnson et al. (2015), although these effects only emerged in a subset of subjects (interoceptive learners). Such marked inter-individual variability may explain the absence of ERP differences between basal and post-feedback interoception in our study, especially since subjects in our sample may have featured varying baseline levels of learning aptitude. Discrepancies with previous studies may also be reflecting methodological differences. For example, while we provided feedback through a stethoscope, Schandry and Weitkunat (1990) offered a feedback tone after each correct response. We surmise that the feedback coming from one's own body may trigger different neural dynamics, though this should be directly assessed in future research.

Notwithstanding, both interoceptive conditions evidenced different functional connectivity patterns. While basal interoception was marked by local frontal connectivity, post-feedback interoception distinctively engaged a fronto-posterior network. This was expected given that both processes involve different mechanism. Interoception relies on attention and perception of internal stimuli, whilst interoceptive learning is a more complex process that includes also the updating of this internal information, but with its integration with previous body signals recall (García-Cordero et al., 2016). In fact, previous fMRI studies support these differences in connectivity patterns. Attention to visceral information is mainly associated with the connectivity of fronto-temporal structures, including connections between ventromedial prefrontal cortex, insula, anterior cingulate cortex, and amygdala (Simmons et al., 2013; Jarrahi et al., 2015; Kleint et al., 2015; García-Cordero et al., 2016; Kuehn et al., 2016). On the other hand, interoceptive learning has been suggested to rely on long-range connections between frontal and temporal areas, such as the inferior frontal gyrus, the parahippocampus, and the hippocampus (García-Cordero et al., 2016). Moreover, interoceptive learning deficits in neuropsychiatric diseases (in particular, Alzheimer's disease) were associated with damage to posterior structures, such as hippocampal and temporal regions, as well as the disconnection of fronto-temporal networks (García-Cordero et al., 2016).

Despite this evidence, none of these studies has accounted for the fast and transient temporal dynamics of interoception [with HEP modulations expected between 200 and 500 ms after the heartbeat (Montoya et al., 1993; Schandry and Montoya, 1996; Pollatos and Schandry, 2004)], given the low temporal resolution of the fMRI technique. Conversely, connectivity analysis based on EEG represent a powerful tool to overcome this temporal limitation and, thus, to evaluate the dynamics of brain networks through the fast coupling of their elements (Cohen, 2011; Barttfeld et al., 2013, 2014; Melloni et al., 2015). A previous work from our group assessed connectivity of post-feedback interoception with EEG but comparing the metacognitive awareness of subjects related to their interoceptive learning skills (Canales-Johnson et al., 2015). The metacognitively congruent group (i.e., those whose verbal report about their performance after the feedback condition was congruent with their real one) presented a denser and more widespread network of post-feedback interoception compared to basal interoception. Although this result aligns with our findings, it does not consider those subjects that might have learnt but claimed that they did not. In this way, our study suggests that—irrespective of the subjects' metacognitive awareness—interoceptive learning is associated with local and widespread networks (Damoiseaux et al., 2006; García-Cordero et al., 2016), thus depending mainly on fronto-posterior long-range connections. This is supported by exteroceptive research showing a role of fronto-posterior networks in the integration of information from learning, memory and multimodal association processes (Squire and Zola-Morgan, 1991).

Compatible results emerged in our connectivity and distance analyses. For basal interoception, connectivity decayed as a function of distance, suggesting the prevalence of local connections. Conversely, post-feedback interoception involved greater connectivity strength in mid- and long-range distances, arguably reflecting the complexity of the underlying learning-based processes and their dependence on distributed connections. Thus, such differences between interoceptive conditions, as revealed by connectivity results, might reflect their distinct levels of complexity beyond the possibilities of scalp-level analyses.

In sum, cardiac interoceptive skills seem sensitive to systematic training. While this effect is not necessarily mirrored by scalp-level HEP modulations, it does appear to imply a reconfiguration of functional connectivity among functionally relevant hubs. In particular, interoceptive learning could be characterized by the establishment of longer, more widespread connections cutting across frontal and temporal regions.

### Clinical implications

The dissociation between attention to internal and external stimuli shown by our HEP results and previous studies (Montoya et al., 1993) is relevant for the assessment of body-brain communication in clinical research. Studies on various psychiatric and neurological conditions have shown alterations in this ERP relative to healthy controls (Terhaar et al., 2012; Muller et al., 2015; Schulz et al., 2015; García-Cordero et al., 2016). However, in all of these studies, HEP results emerged from contrasts between samples with resting-state activity (Muller et al., 2015) or a basal interoceptive condition—i.e., none of them examined differences relative to exteroceptive performance. Thus, such evidence does not show whether the observed modulations reflected alterations in general attentional mechanisms or in specifically interoceptive dynamics. Although further studies are needed, evaluating this modulation might represent a gold-standard approach to assess attentional focus to body sensations. For example, studies of behavioral cardiac perception in panic patients yielded inconclusive results, with patients performing either better (Ehlers and Breuer, 1992) than or similar to controls (Willem Van der Does et al., 2000; Yoris et al., 2015). In this way, if the HEP represents a neural marker of general attention to body states regardless cardiac detection, it might be a more sensible and robust index of potential interoceptive alterations in these patients than cardiac behavioral measures. Future studies may assess whether the attention to bodily signals in panic patients indexed by the HEP might show alterations in its modulation between external vs. internal attention.

## Limitations

Regarding Study 1, the motor component of the interoceptive task selected may have influenced our behavioral and electrophysiological results. However, despite this caveat, note that task employed has been previously validated (Melloni et al., 2013; Couto et al., 2014; Sedeno et al., 2014; Canales-Johnson et al., 2015; Yoris et al., 2015, 2017; García-Cordero et al., 2016) and offers a more precise measure of interoceptive performance than other available paradigms (such as mental or discrimination ones) (Yoris et al., 2015). In addition, in our study, differential HEP modulations between interoception and exteroception emerged from a comparison between two motor-tracking conditions, indicating that they were not driven by motor artifacts.

Regarding Study 2, although intracranial measures provide a unique approach compared to other non-invasive methods, they are only obtained from epileptic patients (Ibanez et al., 2013; Canales-Johnson et al., 2015), who may not represent the healthy population. However, we have controlled this limitation following standard analysis protocols that are detailed in the Data Processing section of Study 2 [i.e., discarding sites that were near to epileptogenic foci or presented epileptogenic activity, establishing stringent inclusion criteria for the remaining channels, and careful inspection of MRI scans to rule out structural abnormalities (Oya et al., 2002; Manning et al., 2009; Hesse et al., 2016)]. In addition, the consistency of results in both subjects emphasizes the robustness of our conclusions and offers insights into the functional dynamics of the human brain (Foster et al., 2015; Hesse et al., 2016).

## Conclusions

This study offers unprecedented spatio-temporal evidence on a neural dissociation between attention to external vs. internal stimuli. In addition, our results indicate that post-feedback interoception might be critically characterized by fronto-temporal widespread connections. The characterization of these different aspects of interoception is relevant as a normative or reference parameter to assess body-brain communication in pathological population.

## Author contributions

IG, AI, and LS designed the study. SE, EM, EH, FB, FA, and MP carried out the experiments. SE, EM, EH, FB, AL, MS, and TB analyzed the experiments. AI, WS, MG, EV, CC, HG, EdH, and FM contributed to the clinic aspects of the paper. IG, LS, AI, and AG wrote the final paper. All authors have approved the manuscript.

### Conflict of interest statement

The authors declare that the research was conducted in the absence of any commercial or financial relationships that could be construed as a potential conflict of interest.

## Abbreviations

HEP: Heartbeat Evoked Potential
M: Mean
SD: standard deviation
HBD task: Heartbeat Detection Task
P1: Patient 1
P2: Patient 2
bpm: beats per minute
EEG: Electroencephalography
ECG: Electrocardiogram
ERPs: Event-Related Potentials
ICA: Independent Component Analysis
wSMI: Weighted Symbolic Mutual Information
BB: Broadband
SEM: Standard error of the mean.
