# Supplementary material for: Attention, in and Out: Scalp-Level and Intracranial EEG Correlates of Interoception and Exteroception
Source: Front Neurosci. 2017 Jul 19;11:411. doi: 10.3389/fnins.2017.00411 (PMC5515904; doi:10.3389/fnins.2017.00411)
Supplement: Supplementary file 1 [file DataSheet1.DOC]

Supplementary Material

**Attention, in and out: Scalp-level and intracranial EEG correlates of interoception and exteroception**

Indira García-Cordero^+^, Sol Esteves^+^, Ezequiel Mikulan, Eugenia Hesse, Fabricio Baglivo, Walter Silva, María del Carmen García, Esteban Vaucheret, Carlos Ciraolo, Hernando Santamaría García, Federico Adolfi, Marcos Pietto, Eduar Herrera, Agustina Legaz, Facundo Manes, Adolfo M. García, Mariano Sigman, Tristán Bekinschtein, Agustín Ibáñez, Lucas Sedeño^*^

+ Indicates equal contribution

*** Correspondence:** Lucas Sedeño: [lucas.sedeno@gmail.com](mailto:lucas.sedeno@gmail.com)

**1 Supplementary Data**

**1.2 Materials and Methods**

**1.2.1 Heartbeat Detection Task**

To assess performance on the HBD Task, we used an index that normalizes the subjects correct responses based on the total amount of heartbeats (Accuracy Index). This index allows us to compare participants’ performance without the bias of heart rate differences. We utilized a modified equation from the one proposed by Schandry; he employs the total amount of mental heartbeats counted and the total number of heartbeats recorded as measures for his index.

Our motor tracking method allows us to discriminate the correct motor response (tapping on the keyboard) of the participants from their incorrect answers. To separate them, every motor response was compared with a specific time window around every recorded heartbeat; if the tapping input was temporarily located within the corresponding time window for each beat, the response was considered as correct (the time window is determined by the subjects’ heart rate: 0.750 milliseconds after the R wave, for a heart frequency less than 69.76; 0.6 after, for frequencies between 69.75 and 94.25; 0.4 milliseconds after, for frequencies higher than 94.25). The total sum of all of the subjects’ responses that fulfilled this temporal criterion was considered to be a correct answer.

Thus, the accuracy equation used was:

1-((Recorded Heartbeats – Total of Correct Answers)/ Recorded Heartbeats))

An accuracy Index was calculated for every condition, varying from 0 to 1, with high scores indicating only small differences between correct answers and recorded heartbeats .

**1.2.2 Functional Connectivity**

The weighted Symbolic Mutual Information (wSMI) measure presents three main advantages . First, it looks for qualitative or “symbolic” patterns of increase or decrease in the signal, which allows a fast and robust estimation of the signals’ entropies. The symbolic transformation depends on the length of the symbols (here, *k* = 3) and their temporal separation (here, τ=16ms between time samples). Second, wSMI makes few hypotheses on the type of interactions and provides an efficient way to detect non-linear coupling. Third, wSMI weights discard the spurious correlations between EEG signals arising from common sources and favor non-trivial pairs of symbols . EEG signals were first transformed into a series of discrete symbols defined by the ordering of k time samples separated by a temporal separation τ. Analysis was restricted to a fixed symbol size (*k* = 3) and a τ=16 ms. Low-pass filters at corresponding frequencies were used to avoid aliasing artifacts. The wSMI was estimated with a joint probability matrix multiplied by binary weights. These weights were set to zero for pairs of (a) identical symbols and (b) opposed symbols that could be elicited by a unique common source or the two sides of a single dipole, respectively.

# Supplementary Table

**Supplementary Table 1.** List of recorded contact sites included in the analysis for each subject. MNI coordinates are reported.

| ***Subject*** | ***Region*** | ***Hemisphere*** | ***Brodmann*** | ***X*** | ***Y*** | ***Z*** |
| --- | --- | --- | --- | --- | --- | --- |
| 1 | Posterior Insula | Right | 48 | 40 | 4 | -8 |
| 1 | Posterior Insula | Right | 48 | 44 | 4 | -8 |
| 1 | Posterior Insula | Right | 48 | 48 | 4 | -8 |
| 1 | Amygdala | Right | 36 | 26 | 2 | -26 |
| 1 | Amygdala | Right | 36 | 30 | 2 | -26 |
| 1 | Amygdala | Right | 36 | 34 | 2 | -26 |
| 1 | SomatosensoryCortex | Right | 2 | 64 | -20 | 32 |
| 1 | SomatosensoryCortex | Right | 2 | 68 | -20 | 32 |
| 2 | Inferior Frontal Gyrus – ParsOpercularis | Left | 48 | -48 | 18 | 10 |
| 2 | Inferior Frontal Gyrus – ParsOpercularis | Left | 48 | -51 | 18 | 10 |
| 2 | Inferior Frontal Gyrus – ParsOpercularis | Left | 48 | -54 | 18 | 10 |

**3 References**

Canales-Johnson, A., Silva, C., Huepe, D., Rivera-Rei, A., Noreika, V., Garcia Mdel, C., et al. (2015). Auditory Feedback Differentially Modulates Behavioral and Neural Markers of Objective and Subjective Performance When Tapping to Your Heartbeat. *Cereb Cortex* 25(11)**,** 4490-4503. doi: 10.1093/cercor/bhv076.

Couto, B., Adolfi, F., Sedeno, L., Salles, A., Canales-Johnson, A., Alvarez-Abut, P., et al. (2015). Disentangling interoception: insights from focal strokes affecting the perception of external and internal milieus. *Front Psychol* 6**,** 503. doi: 10.3389/fpsyg.2015.00503.

García-Cordero, I., Sedeño, L., de la Fuente, L., Slachevsky, A., Forno, G., Klein, F., et al. (2016). Feeling, learning from and being aware of inner states: interoceptive dimensions in neurodegeneration and stroke. *Philosophical Transactions of the Royal Society B: Biological Sciences* 371(1708).

King, J.R., Sitt, J.D., Faugeras, F., Rohaut, B., El Karoui, I., Cohen, L., et al. (2013). Information sharing in the brain indexes consciousness in noncommunicative patients. *Curr Biol* 23(19)**,** 1914-1919. doi: 10.1016/j.cub.2013.07.075.

Melloni, M., Sedeno, L., Couto, B., Reynoso, M., Gelormini, C., Favaloro, R., et al. (2013). Preliminary evidence about the effects of meditation on interoceptive sensitivity and social cognition. *Behav Brain Funct* 9**,** 47. doi: 10.1186/1744-9081-9-47.

Schandry, R., Sparrer, B., and Weitkunat, R. (1986). From the heart to the brain: a study of heartbeat contingent scalp potentials. *Int J Neurosci* 30(4)**,** 261-275.

Sedeno, L., Couto, B., Melloni, M., Canales-Johnson, A., Yoris, A., Baez, S., et al. (2014). How do you feel when you can't feel your body? Interoception, functional connectivity and emotional processing in depersonalization-derealization disorder. *PLoS ONE* 9(6)**,** e98769. doi: 10.1371/journal.pone.0098769.

Yoris, A., Esteves, S., Couto, B., Melloni, M., Kichic, R., Cetkovich, M., et al. (2015). The roles of interoceptive sensitivity and metacognitive interoception in panic. *Behav Brain Funct* 11**,** 14. doi: 10.1186/s12993-015-0058-8.
